# Drinking and Alcohol-Related Problems Among Minority Women

**Published:** 1994

**Authors:** Raul Caetano

**Affiliations:** Raul Caetano, M.D., Ph.D., is the director of the Alcohol Research Group of the California Pacific Medical Center Research Institute, Berkeley, California

## Abstract

Although drinking by black and Hispanic women in the United States differs from that of white women in terms of prevalence rates and incidence of alcohol-related problems, factors such as age and employment status have similar effects on drinking in each group. However, influences on drinking among minority women are complex and must be thought of as an interaction of cultural, personal, and historical factors. This interplay is beginning to emerge from ethnic studies.

This issue of *Alcohol Health & Research World* reviews a representative sample of the spectrum of studies found in the current literature that focus on alcohol consumption and drinking problems in women. Although other studies are available (e.g., Wilsnack and Beckman 1984), few of them assess drinking by ethnic minority women. Leland (1984) reviewed the literature existing for these segments of the population in 1984. At that time, no major survey had been conducted with national samples of U.S. ethnic groups. All large surveys had some analysis of data by ethnicity, but because these surveys were designed to focus on the U.S. general population, the number of minority respondents interviewed as part of their samples was small. The consequent analyses were limited in scope and generaliz-ability, and most were not gender specific.

Since Leland’s review, several surveys have concentrated on drinking in ethnic minority populations. Among them are those that are part of the Epidemiologic Catchment Area studies (ECA), which focused on blacks in St. Louis and Mexican-Americans in Los Angeles; the 1984 National Alcohol Survey (NAS–84) and its 1992 followup, which interviewed national samples of whites, blacks, and Hispanics; and the Hispanic Health and Nutrition Examination Survey (H–HANES 1982–1984), which interviewed a large number of Mexican-Americans, Cuban-Americans, and Puerto Ricans. Studies with a specific focus on women or that reported gender-specific analyses from data collected in these surveys have been published, and some of their findings will be discussed here (e.g., Helzer and Canino 1992; Burnam 1989; Caetano 1991; Herd 1991; Herd and Caetano 1987; Markides et al. 1990).

This article examines data on black and Hispanic women; it also reviews data about white women as a comparison group. These are the three groups of women for which national data are available. Because studies of Asian-Americans and Native Americans are limited to specific localities, they do not represent drinking by women in these two ethnic groups at a national level. Local analyses were used by researchers studying these groups because the great ethnic variation that exists within each group is reflected in their geographical distribution. Local studies are able to include only the Asian national subgroup or the Native American tribe present in the particular locale in which the study is taking place. Grouping these studies to achieve some broader geographic coverage is not warranted because of the differences in methods across studies.

In this article, findings from NAS–84, conducted by the Alcohol Research Group, will be presented along with new findings from the 8-year followup of this sample conducted in 1992. The first part of the article describes the research methodology used in 1984 and 1992, followed by a discussion of findings on drinking patterns and their sociodemographic correlates. A review of drinking problems among black, Hispanic, and white women concludes the article.

## Research Methodology

### Sampling and Data Collection in 1984

A total of 1,947 blacks, 1,453 Hispanics, and 1,777 whites were interviewed for the NAS–84 survey. Subjects were adults selected at random from among individuals living in households in the 48 conterminous United States. The response rates were 76 percent for blacks, 72 percent for Hispanics, and 73 percent for whites.

Data were collected by trained interviewers in face-to-face interviews that lasted an average of 1 hour. The place of interview was the respondent’s home, and the instrument for data collection was a standardized questionnaire. Hispanic respondents were given a choice of being interviewed in English or Spanish—a Spanish version of the questionnaire and bilingual interviewers were assigned when needed. About 43 percent of these respondents chose to be interviewed in Spanish.

### Sampling and Data Collection in 1992

A total of 1,151 blacks, 1,149 Hispanics, and 1,125 whites, who had been interviewed in 1984, were selected for reinterview in 1992, using the following criteria. All respondents who in 1984 reported four or more drinking-related problems (i.e., medical, psychological, or social problems caused or exacerbated by alcohol use) on a lifetime basis and/or reported currently drinking five or more drinks on one occasion were chosen for followup; this garnered 551 blacks, 446 Hispanics, and 619 whites. An additional 600 black, 703 Hispanic, and 506 white respondents also were selected at random to provide a sample of individuals with a wide range of drinking patterns. Out of the contingent selected for reinterview, followup interviews were successfully completed with 723 blacks, 703 Hispanics, and 788 whites, making the overall response rate 70 percent.^1^

### Identifying Participants’ Ethnicity

The main ethnic identifier for sample selection and in the analysis of results in both 1984 and 1992 was the ethnicity of the family of origin. The respondent was asked: “Which of these groups describes your family of origin?” Four categories for identifying blacks, Hispanics, and whites were provided. Respondents who selected the category “black, not of Hispanic origin” were identified as blacks. Respondents who selected “black of Hispanic origin (e.g., Mexican, Central or South American, or any other Hispanic origin)” and “white of Hispanic origin (e.g., Mexican, Central or South American, or any other Hispanic origin)” were classified as Hispanics. Respondents who said that their family of origin was “white, not of Hispanic origin” were identified as white.

This ethnic identification methodology groups a variety of white, black, and Hispanic national groups under all-encompassing ethnic identifying labels. Although cultural diversity exists within each of these larger groups, studies using this general grouping scheme are warranted by the many common social and cultural traits (e.g., language) shared by women in each group.

This article primarily will compare drinking practices and alcohol-related problems among women in the three large ethnic groups established using the method described above. However, it also will examine differences between divisions of the Hispanic group made based on individuals’ nation of origin (i.e., whether they are of Mexican, Puerto Rican, or Cuban origin). In this way, the article will consider some of the intraethnic variation present in the larger Hispanic ethnic group.

### Categorizing Respondents By Alcohol Consumption

Drinkers were placed in categories defining their levels of drinking using a quantity-frequency index. This index is based on the respondent’s self-report of frequency and quantity of drinking wine, beer, and spirits during the 12 months prior to the survey. The respondent’s frequency of drinking was coded in 1 of 11 categories ranging from “never” to “three or more times a day.” Quantity of consumption was assessed by asking for the proportion of drinking occasions on which the respondent drank five or six, three or four, and one or two glasses each of wine, beer, and spirits. This information can be used to classify respondents according to how often they drink five or more drinks of any alcoholic beverage, if they ever do. Cross-tabulating these categories with the frequency of drinking provides the following index categories:

- Frequent heavy drinker: Drinks once a week or more often and has five or more drinks at a sitting, once a week or more often. A drink equals 1 ounce of spirits, a 4-ounce glass of table wine, or a 12-ounce can of beer, each of which contains approximately 12 grams of absolute alcohol.
- Frequent high maximum drinker: Drinks once a week or more often and has five or more drinks at a sitting less than once a week but at least once a year.
- Frequent low maximum drinker: Drinks once a week or more often but never drinks five or more drinks at a sitting.
- Less frequent high maximum drinker: Drinks one to three times a month and has five or more drinks at a sitting occasionally (at least once a year).
- Less frequent low maximum drinker: Drinks one to three times a month but never has five or more drinks at a sitting.
- Infrequent drinker: Drinks less than once a month but at least once a year and may or may not drink five drinks at a sitting.
- Abstainer: Drinks less than once a year or never has consumed alcoholic beverages.

## Drinking Patterns

### Comparisons Among Groups in 1984

In 1984 drinking patterns across white, black, and Hispanic women were different in several ways (table 1). Abstention was higher among black women and Hispanic women than among white women. Approximately half of the black and Hispanic women interviewed reported they had not consumed alcohol in the 12 months previous to the survey. Most women who drank in all three groups were light drinkers (i.e., the majority of them were infrequent drinkers and less frequent low maximum drinkers). The proportion of more frequent drinkers,^2^ such as those who drank at least once a week, was higher among white than among black and Hispanic women in 1984.

Another pattern of drinking—drinking five or more drinks at a sitting—also was more common among white than among black and Hispanic women. This can be seen in table 1 by adding the less frequent high maximum, frequent high maximum, and frequent heavy drinking categories. The sum of these categories shows that 18 percent of the white, 14 percent of the black, and 11 percent of the Hispanic women fall into this combined category.

### Comparing Changes in Drinking Patterns Over Time

Some of the results of the 1992 followup of the 1984 respondents also are shown in table 1. Abstention increased among white and black women but not significantly among Hispanics. Infrequent drinking rose slightly among blacks and Hispanics but not among whites. These two categories are constituted by women who either did not drink or who drank less than once a month in the 12 months prior to the survey. Taken together, 75 percent of the black, 82 percent of the Hispanic, and 58 percent of the white women are in these two categories, attesting to the low levels of drinking reported by women in survey research.

When all the categories are considered, a decrease in drinking between 1984 and 1992 is revealed. The decrease seems to be slightly more pronounced among white women than among black and Hispanic women. Given that women in the sample in 1992 are 8 years older than they were in 1984, and given that drinking decreases with age, reductions in drinking from 1984 to 1992 may have been a result of aging of the sample. However, results from analysis of per capita consumption in the United States (Williams et al. 1992) indicate reductions in alcohol consumption since 1982. Trend analysis on drinking between 1984 and 1990 by Midanik and Clark (in press) indicates that reductions in drinking observed among whites have not been found among blacks and Hispanics, confirming the followup finding in the NAS population.

### Stability of Certain Drinking Patterns Over Time

Table 2 contains other analyses of longitudinal data (i.e., data from participants studied over time) from 1984 through 1992, showing stability for various types of drinking patterns. Stability indicates the proportion of women who had the same drinking pattern in 1984 and 1992 out of all of those who had that drinking pattern in 1984. In table 2, for instance, 81 percent of the 93 white women who were abstainers in 1984 also abstained in 1992.

The results in the table show that abstention is the most stable category in the quantity-frequency index described above, being slightly more constant among white and black women than among Hispanics. This high rate of stability is not surprising, because abstention commonly occurs in the context of strongly held religious beliefs or social norms that decrease women’s access to alcohol and thus decrease their consumption. The other four categories of drinkers in the index show different degrees of stability. White women have a more stable pattern in the categories of drinkers who drink one to three times a month or drink at least once a week; black women have a more consistent group of frequent heavy drinkers who drink five or more drinks at a sitting at least once a week. Longitudinal data (Caetano and Kaskutas 1993) from the NAS for 1984 and 1992 on the mean number of drinks consumed per month show that white and black women who were frequent heavy drinkers had a similar mean consumption in 1984: whites drank 117.2 drinks per person per month; blacks drank 120.5 drinks per person per month. However, in 1992, the mean for white women who were frequent heavy drinkers decreased to 104.0 drinks per person per month, whereas the mean for blacks increased to 148.0 drinks per person per month.

### Comparing Incidence Rates

Table 2 also shows results for incidence rates.^3^ Incidence is the proportion of women who fit into a drinking category in 1992 out of all those who did not fit into that category in 1984. In table 2, for example, of the 308 white women who were not abstainers in 1984, 16 percent reported abstaining from alcohol in 1992. The table indicates that the incidence of abstention is higher among black women than among white and Hispanic women, and the incidence of drinking one to three times per month is higher among white women. Incidence rates for drinking five or more drinks at a sitting or for drinking nearly every day or more are not very different across the three groups of women. In addition, the across-group comparison from 1984 and the followup from 1992 show once again that compared with men, the majority of women in the United States drink little (Caetano and Kaskutas 1993).

### Comparing Prevalence Rates

Within the higher alcohol consumption categories, prevalence rates for frequent heavy drinking are not very different across ethnic groups, but this pattern of drinking is more stable among black than among white women. The mean number of drinks per month consumed by black frequent heavy drinkers is higher than that for whites. These results contradicted previous survey findings (Clark and Midanik 1982), which suggested that black women had both a higher rate of abstention and a higher rate of frequent heavy drinking than did white women. However, the longer career of frequent heavy drinking and higher levels of consumption of black women put them more at risk for developing alcohol problems than whites and Hispanics. These findings also were true for differences between frequent heavy drinking among white, black, and Hispanic men and help explain blacks’ higher rates of medical and other alcohol-related problems when compared with those of whites (Caetano and Kaskutas 1993).

### Examining Differences Among Age Groups

Results by age for the NAS–84 survey (Herd 1988; Herd and Caetano 1987) show that drinking decreases with age among women in all three ethnic groups (table 3). The variation is such that rates of abstention are two times higher among women who are 60 years of age and older than among women 18 to 29 years of age. However, black and Hispanic women have higher rates of abstention than do white women in almost all age groups. When compared with black and Hispanic women in the 18 to 29 and 30 to 39 age groups, white women’s higher drinking rates are concentrated in the less frequent high maximum and frequent high maximum drinking categories.

Only in the middle-age group (ages 40 to 59) do Hispanic drinkers consume more than white drinkers consume. This higher rate of frequent high maximum drinking among middle-age Hispanic women could be explained in terms of an increase in independence among women who are older and have a central role in their family but who also may hold jobs and therefore are able to adopt less traditional roles in the household and in the family.

When analyses compare frequent drinking (drinking once a week or more often) among age groups, results show that such a pattern of consumption is more likely to occur among white women who are younger and who are single. Among black women, however, it is more likely to happen in younger women regardless of marital status (Herd and Caetano 1987). These results for black women contrast with an analysis by Herd (1988), who has reported that black middle-age women rather than younger women were more likely to drink frequently. (These differences in results are most probably linked to the different analytical methods used to examine these data in the two above-mentioned analyses.) Among Hispanic women, those who are younger (18 to 39) are more likely than other women to drink once a week or more often. Factors such as having an annual family income higher than $30,000 and being single also increase the likelihood of a Hispanic woman drinking frequently (Herd and Caetano 1987).

### Income, Education, and Other Sociodemographic Characteristics

The relationship between sociodemographic characteristics, such as income and marital status mentioned above, and drinking is complex and differs across ethnic groups. Among black women, for example, sociodemographic factors seem to have a weaker association with drinking than they do among white women (Caetano and Herd 1984; Herd 1988). It has been suggested that this may be because minority status or cultural characteristics play a larger role in determining drinking by black women, thus weakening the role of income, education, marital status, and other sociodemographic characteristics (Caetano and Herd 1984; Herd 1988).

When analyses focus on differentiating women who drink from those who do not, Herd and Caetano (1987) have shown a direct positive relationship between drinking and income and education for women in all three ethnic groups, although the results for income are weaker among black than among white and Hispanic women. Herd (1988) has added employment status to the analyses, showing that white women are more likely to be drinkers if they are single, have a high income, and are employed; black women are more likely to be drinkers if they are employed. The difference between the initial analyses by Herd and Caetano (1987) and those by Herd (1988) may stem from the inclusion of employment status in Herd’s analyses as well as from differences in statistical methods of analysis.

Further considering these sociodemographic factors, Caetano (1991) found that Hispanic women are more likely to be drinkers if they are acculturated (described below), employed, and educated. Gilbert and colleagues’ (in press) analyses of employment status and drinking among Hispanic women show that professionals have lower rates of abstention and higher rates of frequent drinking (drinking at least once a week) than do women who are homemakers or have blue-collar occupations. Gilbert and colleagues suggest that professional women’s increased use of alcohol may be a way to cope with increasingly complex lives, or it may be a result of their increased exposure to public and private activities in which alcohol is present. An additional factor may be that professionals often have more disposable income that could be spent on alcohol.

### The Influence of Acculturation on Drinking

Acculturation is a complex term that can broadly be understood as the process by which immigrants adopt the norms, social values, and overall culture of the host country. Because drinking norms vary greatly for women among different cultures, acculturation is studied to determine how women’s drinking habits may change as they adapt to a new culture. Acculturation is measured in surveys using a series of questions. In the NAS–84, the acculturation measure was formed from items assessing such attributes as daily use of and ability to speak, read, and write English and Spanish; preference for media in English or Spanish; ethnicity of the people with whom respondents interacted in their church, parties, and neighborhood both at the time of the survey and when growing up; and questions about values thought to be characteristic of the Hispanic way of life.

The acculturation of Hispanics to U.S. society and its effect on alcohol consumption has been the object of several studies (Caetano 1989; Gilbert and Cervantes 1987; Markides et al. 1990; Burnam 1989; Corbett et al. 1991). Although some view acculturation as a stressful process, it may not necessarily be so. The process of adopting the host country’s culture also can be gradual and stress free.

Results of research demonstrate that acculturation is a powerful force shaping women’s drinking patterns. The NAS–84 shows that Hispanic women who are highly acculturated are more likely to drink than are those who are less acculturated. The abstention rate among less acculturated women was 70 percent, whereas in the highly acculturated group, it was 32 percent. In contrast, the proportion of women drinking at least once a week was 22 percent among the highly acculturated but only 3 percent among the less acculturated. The effect of acculturation on drinking and heavier drinking among Hispanic women is independent of the effect of other attributes, such as age, income, education, and being born in the United States.

Acculturation to U.S. society leads to a lower rate of abstention and more light drinking among Hispanic women through several mechanisms. It is associated with more opportunities to drink (Caetano 1987). Hispanic women in the highly acculturated group report a higher frequency of attendance in settings in which alcohol is consumed (e.g., bars and restaurants) than other Hispanic women. Acculturation also may lead to drinking by altering norms and attitudes that regulate alcohol consumption by women. Highly acculturated women have more liberal norms and attitudes toward alcohol consumption than do less acculturated women. For example, U.S.-born Mexican-American women are more likely to see positive effects of alcohol use (e.g., they find it helps them to relax in social situations) (Gilbert 1993) and have more liberal norms and attitudes toward alcohol consumption than do immigrant women (Caetano and Medina Mora 1988). Together, these changes in opportunities to drink, attitudes, and norms regulating alcohol consumption may create an environment that is much more accepting of women’s drinking and thus lead to an increased rate of alcohol consumption among Hispanic women who are acculturated.

### The Influence of Generational Status

Generational status (i.e., whether a woman is U.S.-born or has immigrated to the United States) interacts with acculturation and other sociodemographic factors in shaping Hispanic women’s drinking practices. To understand the way in which drinking is affected by these factors, it is important to separate the effect of acculturation from generational status, because they commonly have identical influences on drinking. Because these two factors produce the same effects and often co-occur in a single person, women who are highly acculturated to U.S. society are more likely to have been born in the United States. However, it is possible that a U.S.-born woman living in a Hispanic community in the United States could be less acculturated than a woman born in a Hispanic country but raised and living in the United States in a multiethnic community.

U.S.-born Hispanic women are more likely to drink alcohol than are immigrant women (Caetano 1987, 1989; Gilbert 1993). Among the U.S.-born women, those of the first generation born in the United States drink more than do second and third generation women. First generation women have a rate of abstention of 22 percent, compared with 30 percent among other Hispanic women (Caetano 1989). Some researchers have interpreted this difference as stemming from conflict between these women and their parents, who bring views on behavior and drinking habits from their country of origin. Likewise, this is thought to be the cause of another pattern of differences in which first generation women have more alcohol-related problems than women in subsequent generations. These characteristics, together with the fact that first generation women have spent their lives in a cultural environment more permissive toward drinking by women, provide some of the reasons for their more widespread use of alcohol when compared with immigrant women.

### Hispanic Drinking: Influences of Specific National Origin

Most U.S. Hispanics identify their cultural heritage as connected with Mexico, Cuba, or Puerto Rico. Data from the 1984 survey showed that Mexican-American women had an abstention rate similar to that of Cuban-Americans (46 percent and 42 percent, respectively) and higher than that of Puerto Ricans (33 percent) (Caetano 1989). However, Mexican-American women had a rate of drinking five or more drinks at least once a week (12 percent) that was higher than that of women in the other two groups (Puerto Ricans, 3 percent; Cuban-Americans, 7 percent).

An analysis by Black and Markides (1993) provided somewhat different findings. They reported that any drinking at all was more common among Puerto Rican and Mexican-American women than among Cuban-Americans. However, national origin did not change the effect of acculturation on drinking described previously. Black and Markides (1993) showed that Mexican-American, Cuban-American, and Puerto Rican women who were more highly acculturated were more likely to be drinkers and also more likely to have a higher frequency of alcohol consumption than were women at lower acculturation levels.

## Alcohol-Related Problems

Because they drink less, women have a lower prevalence of alcohol-related problems than do men. This fact has led to more detailed research and analysis of women’s drinking and heavy drinking than of their alcohol problems, especially for minority women. The only survey with national data on problems among black and Hispanic women is the NAS–84, which collected prevalence data on 14 alcohol-related problems from white, black, and Hispanic women (Herd and Caetano 1987; table 4). In general, the proportion of white women who reported alcohol-related problems was higher than the proportion of blacks and Hispanics reporting them. However, the differences were not greater than 3 percentage points in magnitude. When these data were examined for drinkers only (results not shown), the rates in table 4 became slightly higher, but white women still reported more problems than did blacks and Hispanics. This demonstrates that the difference in problem rates across the three ethnic groups was not caused by an increased rate of drinking among white women.

The most frequently reported problems across women in the three groups were salience of drinking (i.e., when other aspects of life take a secondary place to drinking); belligerence; health problems; and other drinking-related problems, such as arguments with people other than the spouse. When these problems were counted and a summary was constructed, the proportion of white, black, and Hispanic women reporting one or more alcohol-related problems was 12 percent, 7 percent, and 6 percent, respectively (Herd and Caetano 1987).

### Examining Predictors of Alcohol-Related Problems

Comparing factors that predict alcohol-related problems among white, black, and Hispanic women revealed that white and black women with less education; who were single, separated, or divorced; and who were younger were more likely to report problems than were other women (Herd and Caetano 1987).

Among Hispanic women, the factors associated with a greater likelihood of reporting alcohol-related problems included being married, being young, and being highly acculturated. An additional factor associated with reporting problems among Hispanic women was being born in the United States and having at least one parent who also was U.S. born. This may be explained by these women having the highest rate among Hispanic women of drinking at least once a week and also drinking five or more drinks per occasion at least once a week.

Among Mexican-American women, those who were high in acculturation and those who were single, separated, or divorced were more likely to have alcohol-related problems than were other women. However, when drinking patterns were included in these analyses with Mexican-American women, being a more frequent drinker (drinking at least once a week and drinking five or more drinks per occasion at least once a year) was the only characteristic associated with problems.

### Prevalence of Alcohol Dependence Among Ethnic Groups

Rather than examining problems per se, other studies have assessed the prevalence of alcohol abuse and dependence as defined in the *Diagnostic and Statistical Manual of Mental Disorders, Third Edition* (American Psychiatric Association 1980). These definitions require the presence of alcohol-related problems (e.g., withdrawal, impairment of control over alcohol consumption, social or occupational impairment because of use of alcohol) for a positive diagnosis.

Reports based on the Baltimore, St. Louis, and North Carolina general population samples from the ECA study indicated that the 12-month prevalence rate for alcohol abuse and dependence among white and black women was 1 percent and 2 percent, respectively (Robins 1989).

Data by age from the five samples in the ECA study revealed a different pattern in the prevalence of alcohol abuse and dependence across the two groups of women. Among white women, prevalence was higher in the 18 to 29 age group, decreasing continuously in older age groups. Among black women, prevalence rose from the 18 to 29 to the 30 to 44 age groups, remained high in the 45 to 64 age group, and decreased among women 65 years of age and older (Helzer and Canino 1992). These results reproduce the pattern found among men in the same groups for both alcohol abuse and dependence and for all alcohol-related problems.

Several factors could be behind these differences. Cultural differences could be related to access to alcohol by different sets of ages across the two ethnic groups. Also, drinking by women in each group could reflect men’s drinking patterns (i.e., the drinking done by women’s male companions, husbands, or boyfriends).

Burnam (1989) and Canino and colleagues (1992) have reported rates of alcohol abuse and dependence for a sub-population of Hispanics consisting of Mexican-American women in the Los Angeles sample of the ECA study. Comparison of Mexican-American women with white women showed that white women had higher rates of lifetime alcohol abuse and dependence (9 percent versus 4.5 percent), but when the effect of education was controlled in the analysis, the difference disappeared. When data on each dependence problem per se were considered, white women had higher rates than did Mexican-American women for 16 of the 24 alcohol-related problems in the survey. Data by generational status showed that the rate for lifetime prevalence of alcohol abuse and dependence was 2 percent among immigrant Mexican-American women and 10 percent among U.S.-born Mexican-American women. All the Mexican-American immigrant women with a positive diagnosis of abuse and dependence were in the 18 to 24 age group. Among U.S.-born women, the prevalence was higher for younger women (12 percent among those 18 to 44 years of age), but older women also qualified for a diagnosis.

## Discussion

### Explaining the Trends

The results discussed above, especially those that emerged from analyses of the 1984 survey, show drinking by black and Hispanic women in a new light. Previous studies had put forward explanations of drinking by black and Hispanic women, most of which relied too heavily on a central assumption about the groups’ cultures. Drinking by black women was thought to be an effect of “matriarchy”—that it was related to the role of head-of-household and breadwinner forced on black women by the disintegration of the black family. Abstention and drinking by Hispanic women was explained by “marianismo,” which sees women as the center of family life, a vision that demands chastity, purity, and abstention from alcohol.

More recently, explanations of minority drinking have placed considerable emphasis on use of alcohol to minimize stress related to immigration, acculturation, poverty, racial discrimination, and powerlessness. These indeed are powerful constants in the life of minorities in the United States. However, many of these explanations do not actually assess levels of stress among minorities but assume that processes such as acculturation or discrimination increase stress, which then leads to drinking.

### Drinking Associated With Normal Life Changes

Drinking also occurs because of ordinary adaptations to changes in norms, attitudes toward drinking, and an increase in disposable income. These changes may come about because of acculturation, immigration, or changes in job status. Also, it is important to acknowledge the existence of some resolutions to stressful situations associated with acculturation or racial discrimination that are healthy and that do not involve the adoption of deviant or pathological behavior. Emphasis on unhealthy solutions limits understanding of the range of coping mechanisms minorities have. Theories based on a single concept are too simplistic to provide an accurate explanation for the drinking behavior of black and Hispanic women.

### Drinking in the Real World

The reality of minority women’s lives is not necessarily one dominated by a series of stressful changes or by overriding definitions of their role in their cultures. It is more complex and thus requires more complex explanations. The literature reviewed in this article suggests that to understand drinking by black and Hispanic women, one must take into account an interplay of cultural, historical, and socioeconomic factors that is just beginning to emerge from ethnic studies.

Herd (1985) has demonstrated how historical factors, such as the migration of blacks from the rural areas of the South to the industrial cities of the Northeast, influenced black drinking. Findings from studies examining the effects of generational status and acculturation among Hispanic women have shown that these are powerful forces shaping drinking in this group. Sociocultural and historical influences such as these interact with personal factors, such as age and employment status, and larger environmental characteristics, such as the time and place where drinking occurs, to determine the type of alcohol consumption that takes place and its consequences. Analysis by Wilsnack and colleagues (1987) provides a good example of the complexity of factors that influence women’s drinking and problems.

On a more individual scale, women now have diverse societal roles that help determine their opportunities to drink, the amount of money they have to spend on alcohol, and whether those close to them will accept their drinking. Some of these factors have been reviewed by Wilsnack and Wilsnack (in press). Women also are influenced through their recognized role as caretakers or by men’s drinking (i.e., drinking by male partners or companions). Often the alcohol-related problems affecting women’s lives are those associated with the drinking habits of their fathers, brothers, husbands, and boyfriends.

Environmental factors affecting the availability of alcohol in the community also have to be considered when trying to understand drinking by minority women. Some of these factors include alcoholic beverage prices, advertisements, production, and marketing. The minority-directed advertisement of alcoholic beverages as well as the production of special brands of alcoholic beverages targeting minority drinkers have been the subject of much controversy. Several critics argue that minority neighborhoods have an excessive number of outdoor advertisements and liquor stores, some of which are the focus of crime and drinking by minors. By increasing alcohol availability and the acceptability of drinking in minority communities, these factors could lead to increased drinking and increased incidence of alcohol problems among minority women.

Future research on drinking and alcohol problems among minority women should take this complex web of factors into consideration. The possibility that different types of drinking have different determinants also must be taken into account. It is possible that abstention and light drinking are more determined by cultural, social, and historical characteristics than are heavier patterns of drinking, which lead to alcohol abuse and dependence. Personality characteristics and women’s personal and family histories may be of importance in the development of these pathological forms of drinking.

Updated survey research focusing on minority women is needed. The NAS–84 is now 10 years old, and more recent data are necessary to provide a current view of drinking by ethnic minority women. Beginning in 1995 the Alcohol Research Group will conduct a survey updating the information from 1984 and allowing for trends analyses between 1984 and 1995.

Unfortunately, this survey will not focus on Asian-American and Native American women. National studies with a focus on these two groups of women are particularly scarce and should be conducted in the near future. Clinical studies of the effectiveness of alcoholism treatment and access to treatment with representative samples of minority women also must be executed. Until such research has been completed, highly productive alcoholism treatment programs are unlikely to be developed for these populations.

## Figures and Tables

**Table 1:** Drinking Patterns Among White, Black, and Hispanic Women: 1984 and 1992 (in percent)^1^

	White (*n* = 399)		Black (*n* = 402)		Hispanic (*n* = 374)	
			
Drinking Pattern	1984	1992	1984	1992	1984	1992
Abstention	31	36	46	51	47	48
Infrequent	23	22	18	24	27	34
Less Frequent Low Maximum	6	13	18	15	9	11
Less Frequent High Maximum	6	6	6	4	3	4
Frequent Low Maximum	15	12	9	4	4	3
Frequent High Maximum	8	3	4	4	7	2
Frequent Heavy	4	3	4	5	1	3

1 Sample sizes are unweighted. Percentages are weighted (i.e., they have been adjusted to allow for differences in sampling techniques).

NOTE: In this table, Chi^2^ (χ^2^) tests provide an indication of how likely it is that the observed differences between 1984 and 1992 occurred by chance alone:

χ^2^ = Whites 1984 × 1992 = 18,263; degrees of freedom (df) = 6; *p* < 0.01

χ^2^ = Blacks 1984 × 1992 = 18,467; df = 6; *p* < 0.01

χ^2^ = Hispanics 1984 × 1992 = 19,560; df = 6; *p* < 0.01.

**Table 2:** Stability and Incidence of Selected Drinking Patterns Among White, Black, and Hispanic Women: 1984 and 1992 (in percent)^1^

	Stability				Incidence			
		
Drinking Pattern	Year	White	Black	Hispanic	Year	White	Black	Hispanic
Abstains	1992	81%	81%	71%	1992	16%	26%	13%
	1984	(93)^2^	(151)	(188)	1984	[308]^3^	[257]	[189]
Drinks 1 to 3 times per month	1992	49%	21%	19%	1992	18%	10%	10%
	1984	(93)	(81)	(68)	1984	[308]	[327]	[309]
Drinks once a week or more	1992	50%	46%	25%	1992	5%	6%	5%
	1984	(135)	(109)	(52)	1984	[266]	[299]	[325]
Drinks 5 or more drinks at least once per week	1992	19%	33%	-0-	1992	2%	4%	3%
	1984	(31)	(31)	(11)	1984	[371]	[379]	[367]
Drinks nearly every day or more	1992	32%	31%	-0-	1992	3%	3%	1%
	1984	(30)	(25)	(5)	1984	[371]	[383]	[372]

1 Sample sizes are unweighted. Percentages are weighted (i.e., they have been adjusted to allow for differences in sampling techniques).

2 Under stability, the numbers in parentheses represent the total number of women who reported the drinking pattern category in 1984. For example, 81% of the 93 white women who were abstainers in 1984 also abstained in 1992.

3 Under incidence, the numbers in brackets represent the total number of women *who did not report* the drinking pattern category in 1984. For example, of the 308 white women who were not abstainers in 1984, 16% reported abstaining from alcohol in 1992.

**Table 3:** Drinking Patterns by Age (in years) Among White, Black, and Hispanic Women: 1984 National Alcohol Survey (in percent)^1^

	18–29			30–39			40–49			50–59			60+		
					
Drinking Pattern	White (*n* = 236)	Black (*n* = 396)	Hispanic (*n* = 280)	White (*n* = 217)	Black (*n* = 232)	Hispanic (*n* = 231)	White (*n* = 144)	Black (*n* = 141)	Hispanic (*n* = 129)	White (*n* = 121)	Black (*n* = 129)	Hispanic (*n* = 87)	White (*n* = 311)	Black (*n* = 179)	Hispanic (*n* = 115)
Abstention	22	34	40	30	32	45	35	56	41	35	60	47	49	69	78
Infrequent	20	19	35	16	19	23	21	11	19	21	14	8	18	12	11
Less Frequent Low Maximum	14	22	6	16	18	18	12	11	5	12	12	11	15	8	5
Less Frequent High Maximum	13	4	6	10	6	5	6	5	17	4	2	3	1	2	0
Frequent Low Maximum	11	9	2	7	15	3	13	7	2	22	7	4	15	8	6
Frequent High Maximum	13	6	8	13	5	4	7	4	14	4	4	20	1	0	0
Frequent Heavy	7	6	2	8	5	2	7	6	2	1	2	8	1	1	0

1 Sample sizes are unweighted. Percentages are weighted (i.e., they have been adjusted to allow for differences in sampling techniques).

1 Sample sizes are unweighted. Percentages are weighted (i.e., they have been adjusted to allow for sampling techniques).

**Table 4:** Alcohol-Related Problems Among White, Black, and Hispanic Women: 1984 National Alcohol Survey (in percent)^1^

Problem	White (*n* = 1,029)	Black (*n* = 1,204)	Hispanic (*n* = 842)
Belligerence	5.7	2.5	3.3
Health Problems	4.8	4.3	3.0
Salience of Drinking	4.6	3.8	3.1
Impaired Control	3.7	2.8	2.5
People Problems	3.5	2.0	3.3
Spouse Problems	3.4	2.4	1.1
Withdrawal Symptoms	2.9	1.8	0.9
Tolerance	1.8	0.7	0.3
Financial Problems	1.7	0.6	0.2
Job Problems	1.0	0.7	0.2
Police Problems	0.7	0.5	0.9
Binge Drinking	0.6	0.5	0
Craving	0.5	0.5	0.3
Accident	0.4	0.3	0

1 Sample sizes are unweighted. Percentages are weighted (i.e., they have been adjusted to allow for differences in sampling techniques).

## Footnotes

1 The response rate varied from 75 percent among whites to 68 percent among blacks and 63 percent among Hispanics. Analyses in this article have been performed on weighted data, which correct the figures for the oversampling of heavier drinkers as well as for nonresponse and differences in the probability of selection into the sample. Comparative analysis of 1984 data between the sample reinterviewed and the sample selected for interview (respondents and nonrespondents) yielded no significant differences in the distribution of drinking problems (the mean number of drinks consumed per month or the number of alcohol problems present in the past 12 months).

2 This type of drinking is represented in the quantity-frequency category index by the categories “frequent low maximum” and “frequent high maximum” drinking.

3 Incidence is defined as the number of new cases of a specific condition, in this case of a drinking category, that occur during a certain period. Prevalence is the number of cases of a condition that are present in a population at one point in time.
